# Smoking Cessation therapy is a cost-effective intervention to avoid tooth loss in Brazilian subjects with periodontitis: an economic evaluation

**DOI:** 10.1186/s12903-021-01932-2

**Published:** 2021-12-03

**Authors:** Maria Luisa Silveira Souto, Fernanda Campos de Almeida Carrer, Mariana Minatel Braga, Cláudio Mendes Pannuti

**Affiliations:** 1 Division of Periodontics, University of São Paulo, School of Dentistry, Av. Prof. Lineu Prestes, 2227, São Paulo, SP 05508-000 Brazil; 2 Division of Social Dentistry, University of São Paulo, School of Dentistry, São Paulo, Brazil; 3 Division of Pediatric Dentistry, University of São Paulo, School of Dentistry, São Paulo, Brazil

**Keywords:** Cigarette smoking, Smoking cessation, Periodontitis, Health economics, Modelling

## Abstract

**Background:**

Smokers present a higher prevalence and severity of periodontitis and, consequently, higher prevalence of tooth loss. Smoking cessation improves the response to periodontal treatment and reduces tooth loss. So, the aim of this study was to evaluate the efficiency in resources allocation when implementing smoking cessation therapy vs. its non-implementation in smokers with periodontitis.

**Methods:**

We adopted the Brazilian public system perspective to determine the incremental cost-effectiveness (cost per tooth loss avoided) and cost-utility (cost per oral-related quality-adjusted life-year ([QALY] gained) of implementing smoking cessation therapy. Base-case was defined as a 48 years-old male subject and horizon of 30 years. Effects and costs were combined in a decision analytic modeling framework to permit a quantitative approach aiming to estimate the value of the consequences of smoking cessation therapy adjusted for their probability of occurrence. Markov models were carried over annual cycles. Sensitivity analysis tested methodological assumptions.

**Results:**

Implementing the therapy saved approximately US$ 100 over the time horizon accompanied by a slightly better effect, both in CEA and CUA. Considering uncertainties, the therapy could be cost-effective in the most part of simulated cases, even being cheaper and more effective in 35% of cases in which the oral-health related outcome is used as effect. Considering a willingness-to-pay of US$100 per health effect, smoking cessation therapy was cost-effective, respectively, in 72% and 99% of cases in cost-utility and cost-effectiveness analyses.

**Conclusions:**

Implementation of smoking cessation therapy may be cost-effective, considering the avoidance of tooth loss and oral health-related consequences to patients.

**Supplementary Information:**

The online version contains supplementary material available at 10.1186/s12903-021-01932-2.

## Background

Cigarette smoking is a global public health problem associated with high morbidity and mortality [1]. It is a major risk factor for health problems, such as cancer, cardiovascular and respiratory diseases. Besides, smoking is a risk factor for oral diseases, such as oral cancer, periodontitis, gingival recession, tooth loss and implant failure [2–4]. Smoking is also associated with higher costs in periodontal treatment [5–7] and increases the cost of life-time periodontal treatment from 8.8% up to 71.4% [7].

There is overwhelming evidence about the benefits of smoking cessation to general health [8, 9]. Quitting smoking also improves oral health conditions. Two interventional studies observed greater probing depth reduction and clinical attachment gain in periodontitis patients that quit smoking when compared to non-quitters [10, 11]. Moreover, observational studies have shown that former smokers lose fewer teeth than current smokers [12, 13]. Therefore, smoking cessation therapy (SCT) is recommended as an important component of periodontal treatment [14].

Medical literature demonstrated that SCT is cost-effective because it reduces health care expenditures associated with the effects of smoking [15, 16]. There are some economic evaluations of periodontal therapy in the literature [17–20]. However, to the extent of our knowledge, no study has evaluated the cost-effectiveness of the implementation of smoking cessation therapy for periodontitis patients. When allocating resources, different sources of resources may be considered when considering For a given budget (e.g. those specifically assigned to the oral health department or section) and considering an acceptable outcome (tooth loss), economic evaluations may support an intervention that results in any improvement (in this case, in oral health) that may justify an optimal reallocation of health care resources [21]. Otherwise, lack of data about these consequences may prevent such type of reallocation in real life [21].

We hypothesized that SCT is a cost-effective intervention because it reduces the risk of tooth loss and consequentlyreduces the costs associated with therapies aimed to replace teeth (prosthesis and implants). Such appraisal could be extremely relevant in decision-making since smoking cessation therapy is available in the Brazilian public health system (PHS), but is underused by dentists. Additionally, it may contribute to direct the need of reallocation of resources in oral health care, reinforcing the resources allocation focused on general health. Therefore, this study aimed to evaluate the efficiency in resource allocation when implementing smoking cessation therapy (SCT) vs. its non-implementation in smokers with periodontitis that received periodontal treatment to prevent tooth loss, in the context of the Brazilian PHS.

## Methods

This economic evaluation has been prepared according to the Consolidated Health Economic Evaluation Reporting Standards (CHEERS) [22].

### Setting and model

This study describes a decision-analytic model considering a 48 years-old male Brazilian subject as the base-case. The life expectancy of a 48 years-old Brazilian male is 28 years [23]. Therefore, the time horizon of the analysis was set at 30 years.. In this model, subjects are tobacco smokers with high level of dependence (> 10 cigarettes per day), with 20 teeth and generalized periodontitis stage III, grade C [24, 25]. Age and number of remaining teeth were based on a previous smoking cessation cohort from our group [26].

The study was conducted from the Brazilian PHS perspective. Data were modeled using a Markov simulation model. Tree Age Pro 2017 (TreeAge Software, Williamstown, MA, USA) was used for data modeling and analysis.

### Comparators

A decision-analytic modeling framework was constructed to estimate the efficiency in resource allocation when implementing SCT in smoker patients with periodontitis that received periodontal treatment to prevent tooth loss (Additional file 1).

Subjects entering the model were smokers with periodontitis. Some subjects were supposed to receive SCT and others not. In both cases, they could stop smoking or not. All subjects received non-surgical periodontal treatment (six sessions of scaling and root planning) and one session of maintenance therapy (one session of scaling and root planning). In the decision-analytic modeling framework, this stage was represented as a simple decision tree. We did not consider the possibility of relapsing smoking and/or need of reintervention with SCT.

**Fig. 1:**
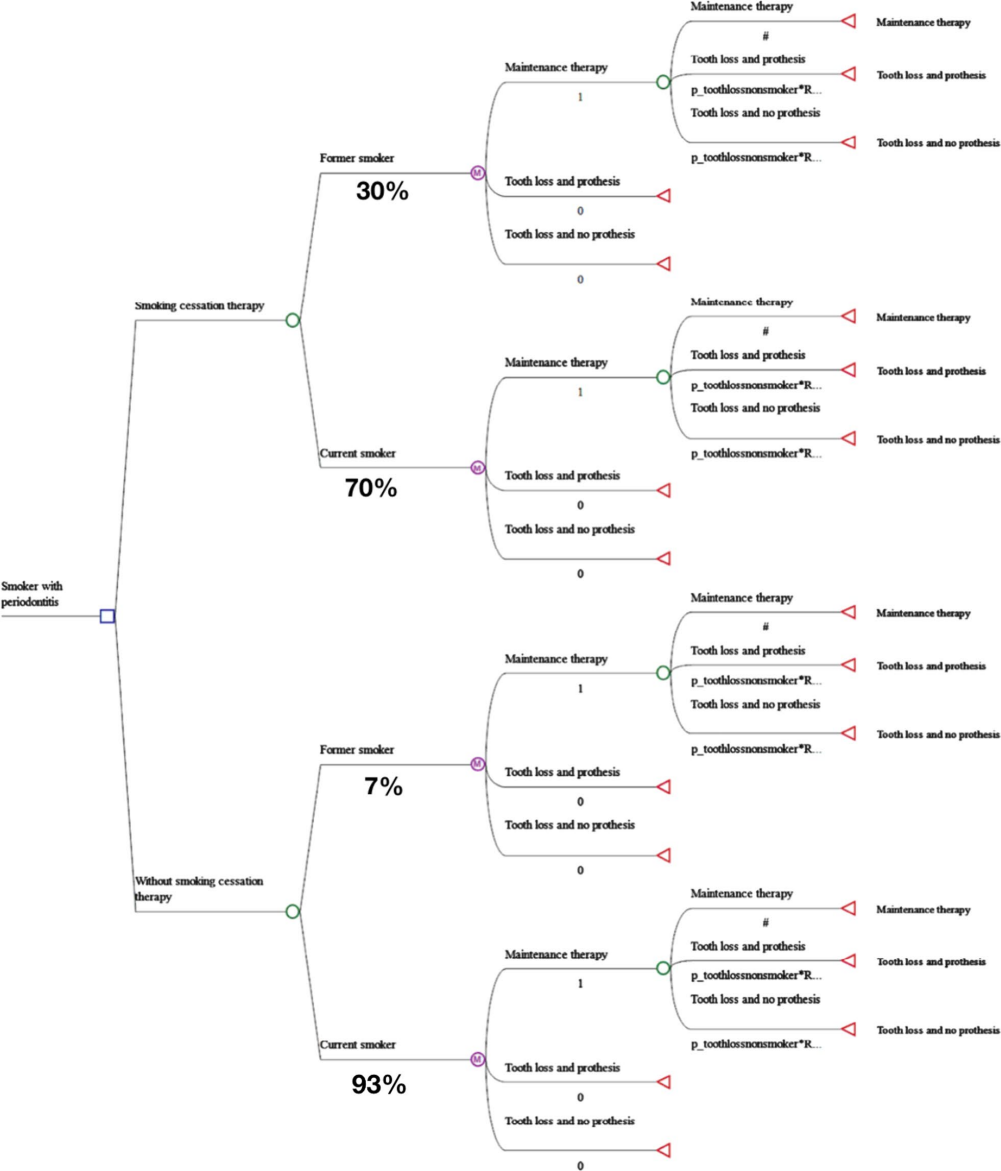
Decision tree: states and transitions used in the model

In the Markov models, at each cycle, we considered that patients could transit among possible health statuses. Thus, after initial treatment, at the end of each year (cycle), subjects with periodontitis could transit between three states: 1) to stay in maintenance therapy with no tooth loss, 2) to lose a tooth and not receive rehabilitation, or 3) to lose a tooth and receive prosthetic rehabilitation. When a tooth loss occurred, we assumed that the remaining teeth would continue to receive periodontal maintenance therapy (Fig. 1; Additional file 1). As in a previous paper [7], we assumed that current smokers would require two extra sessions of maintenance therapy. Further, we assumed that quitters would present a better response to periodontal therapy [26]. To avoid clustering effect, only one tooth per patient was simulated.

### Discount rate

To adjust time preference, costs and benefits were adjusted. We considered a 5% discount rate in this model, following Brazilian guidelines [27].

### Modeled parameters

Transition probabilities were defined by data from previous published studies. Since we used annual cycles in the model, if a study used a different time frame (e.g., 10 years), we converted this probability in a 1-year value (Table 1).

**Table 1:** States and Probabilities used in the model

State	Probability	Data Source
Smoking cessation with therapy	0.3	Prado et al. [28]
Smoking cessation without therapy	0.07	Zhu e al. [29]
Tooth loss in non-smokers	0.003	Fardal et al. [60]
Tooth loss in former smokers	1.15^a^	Souto et al. [13]
Tooth loss in current smokers	2.16^a^	Souto et al. [13]
No rehabilitation	0.09	Pereira et al. [30]

^a^Risk ratio of tooth loss

The probability of quitting smoking with SCT after one year was 30% [28], and the probability of quitting smoking without any therapy was 7% [29].

The probability of tooth loss during maintenance therapy for former and current smokers was calculated using risk ratios of tooth loss in current and former smokers [13, 30], according to the expression below:

$${\text{p}} = {\text{RR}}_{{{\text{tooth}}\,{\text{loss}}}}^{*} {\text{X}}\,{\text{p}}_{{{\text{tooth}}\,{\text{loss}}\,{\text{non - smokers}}}}$$

In our model, when the tooth was lost, the subject could receive rehabilitation with partial prosthesis or could not receive any rehabilitation. To obtain the pooled probability of tooth loss among the non-smokers we gathered individual data from the meta-analysis (when it was available) and performed a meta-analysis of 1-year probabilities (Additional file 3).

### Health effects

The cost-effectiveness analysis (CEA) considered the presence of the tooth (maintenance therapy) as treatment success and tooth loss as a failure, regardless of prosthetic rehabilitation or not.

The cost-utility analysis (CUA) combined utility values with time spent in a state of health, resulting in the number of quality-adjusted life years (QALY) [31]. We determined utilities converting Oral Health Impact Profile (OHIP) scores [32, 33] to a continuous value from 0 (worst oral health state imaginable) to 1 (best oral health state imaginable). We selected OHIP values from studies with Brazilian subjects. We assumed a linear relationship between OHIP scores and the oral health related utility scores obtained. Utility scores with corresponding OHIP score and data source are shown in Additional file 2.

### Outcomes

We calculated incremental costs (Δcost) and incremental effects (Δeffect) based on the assumption that incremental cost per health effect could be estimated to determine the differences both in costs and in the effects of the implementation of SCT, over non-implementation.

### Costs and resources

We used costs from the Brazilian public service. We considered only direct costs of the procedures (Table 2) and the need for one cycle of SCT. Possible relapses and need for repetition of SCT were not explored in this study.

**Table 2:** Cost survey on several states

Included costs	Cost (R$)	Cost (US dollar)^a^	Data Source
**Smoking cessation therapy**			
Cognitive therapy, nicotine replacement therapy (patches 7, 14 or 21 mg and gums 2 or 4 mg) and bupropion 150 mg	559.43	227.41	Mendes et al. [34]
Total	559.43	227.41	
**Periodontal treatment**			
Periodontist hourly wages (06 sessions)	200.10	81.34	Oliveira et al. [35]
Scaling and root planning (06 sessions)	188.58	76.66	Portaria n^o^ 1.464, 24 de junho de 2011
Periaphical radiographs	24.50	9.96	
Maintenance therapy (scaling and root planning + periodontist hourly wages)	64.78	26.29	Oliveira et al. [35]// Portaria n^o^ 1.464, 24 de junho de 2011
Total	477.96	194.25	
**Maintenance Therapy for former smokers**			
Periodontist hourly wages (02 sessions/year)	66.70	27.11	Oliveira et al. [35]
Scaling and root planning (02 sessions/year)	62.86	25.55	Portaria n^o^ 1.464, 24 de junho de 2011
Total	129.56	52.66	
**Maintenance Therapy for current smokers**			
Periodontist hourly wages (04 sessions/year)	133.40	54.23	Oliveira et al. [35]
Scaling and root planning (04 sessions/year)	125.72	51.11	Portaria n^o^ 1.464, 24 de junho de 2011
Total	259.12	105.33	
**Tooth extraction and not rehabilitate**			
Dentist hourly wages (01 session)	33.35	13.56	Oliveira et al. [35]
Analgesic 06/06 h for 3 days	0.6	0.24	BPS – Health Price Bank
Anti-inflammatory 12/12 h for 3 days	0.36	0.15	BPS – Health Price Bank
Total	34.31	13.95	
**Tooth extraction and partial removable prosthesis**			
Dentist hourly wages (05 sessions)	166.75	67.78	Oliveira et al. [35]
Temporary prothesis	24.14	9.81	PHS unified table—SIGTAP
Partial removable prosthesis	150	60.98	PHS unified table—SIGTAP
Panoramic radiograph	9.03	3.67	PHS unified table—SIGTAP
Total	349.92	142.24	
**Tooth extraction and implant**			
Dentist hourly wages (06 sessions)	200.10	81.34	Oliveira et al. [35]
Implant	260.10	105.73	PHS unified table—SIGTAP
Prothesis	300	121.95	PHS unified table—SIGTAP
Analgesic 06/06 h for 3 days	0.6	0.24	BPS—Health Price Bank
Anti-inflammatory 12/12 h for 3 days	0.36	0.15	BPS—Health Price Bank
Panoramic radiograph	9.03	3.67	PHS unified table—SIGTAP
Periaphical radiograph	3.5	1.42	PHS unified table—SIGTAP
Cone-Beam Tomograph	86.75	35.26	PHS unified table—SIGTAP
Total	860.44	349.77	

^a^US dollar was converted by purchasing power parity (PPP). Conversion 2.46. International Monetary Fund. Available from:https://www.imf.org/external/datamapper/PPPEX@WEO/OEMDC/ADVEC/WEOWORLD. Cited 12 Marc 2019

SCT adopted in Brazil consists of cognitive-behavioral therapy and pharmacotherapy. The Brazilian SCT model consists of four weekly sessions in the first month and 12 sessions until one year of treatment. The therapy is conducted by a multidisciplinary team comprising physicians, nurses and psychologists, in specialized centers. Pharmacotherapy consists of nicotine replacement therapy (NRT) and bupropion hydrochloride (150 mg). NRT is based on the combined use of transdermal nicotine patches (7, 14 or 21 mg according to the daily number of cigarettes), and nicotine gum (2 or 4 mg according to the daily number of cigarettes). Brief counseling is conducted during routine consultations. We based the direct costs on a trial conducted in Brazil [34]. Costs with professional training for SCT were not considered in the primary model, but they were modeled in the sensitivity analysis. In the Brazilian PHS, periodontal treatment, maintenance therapy and prosthetic rehabilitation are conducted in Dental Specialty Centers (CEOs). We chose partial removable prosthesis as the type of prosthetic rehabilitation in our model because partial fixed prosthesis is not performed in the Brazilian PHS. Implant therapy was modeled in the sensitivity analysis, since this therapy is performed only in some CEOs, in some Brazilian cities.

We calculated the costs of periodontal procedures, prosthetic rehabilitation and implant therapy considering the hourly wages of dentists from the Dental Specialty Centers (CEOs) [35], the PHS unified table and the costs of the medications (BPS- Health Price Bank) [36].

All costs from years before 2021 were adjusted for inflation using the National Consumer Price (https://www.bcb.gov.br/acessoinformacao/calculadoradocidadao). The conversion into purchasing power parity (ppp) was based on The International Monetary Fund considering 1.00 US dollar as R$ 2.46 (https://www.imf.org/external/datamapper/PPPEX@WEO/OEMDC/ADVEC/WEOWORLD).

### Sensitivity analysis

Deterministic and probabilistic sensitivity analyses were conducted considering the figures shown on Table 3. Time horizon and model settings were kept constant.

**Table 3:** States and probabilities used in sensitivity analysis

Deterministic sensitivity analysis			
State	Minimum value	Base value	Maximum value
Cost of smoking cessation therapy	185.86	227.41	525.59
Cost of smoking cessation therapy (plus maintenance cost^b^)	271.77	227.41	611.50
Cost of periodontal treatment	174.82	194.25	213.68
Cost of maintenance therapy for current smokers	94.80	105.33	115.86
Cost of maintenance therapy for former smokers	47.39	52.66	57.93
Cost of not rehabilitating a tooth loss	12.56	13.95	15.35
Cost of rehabilitating a tooth loss	128.02	142.24	156.46
Discount rate	0.03	0.05	0.07
Probability of smoking cessation with some therapy	0.16^c^; 0.23	0.30	0.38
Probability of tooth loss in non-smokers	0.0003	0.003	0.005 (BR)- 0.006#0.09 (WW)
Probability of losing a tooth and not rehabilitate	0.09	0.09	0.41
Utility periodontitis	0.67	0.76	0.89
Utility after periodontal treatment	0.76	0.86	0.96
Utility maintenance therapy	0.85	0.93	0.98
Utility of tooth loss and no rehabilitation	0.55	0.61	0.67
Utility tooth loss and removable partial prothesis	0.62	0.69	0.76
Risk of tooth loss for former smokers	0.98	1.15	1.35
Risk of tooth loss for current smokers	2.29	2.60	2.96, 4.17^c^

Probabilistic sensitivity analysis			
State	Distribution	Minimum value	Maximum value
Cost of smoking cessation therapy	Triangular	185.86	525.59
Cost of periodontal treatment	Triangular	174.82	213.68
Cost of maintenance therapy for current smokers	Triangular	94.80	115.86
Cost of maintenance therapy for former smokers	Triangular	47.39	57.93
Cost of tooth extraction and not rehabilitate	Triangular	12.56	15.35
Cost of tooth extraction and rehabilitate	Triangular	128.02	156.46
Discount rate	Uniform	0.03	0.07
Probability of smoking cessation with therapy	Triangular	0.23	0.38
Probability of tooth loss in non-smokers	Triangular	0.0007	0.0127
Probability of losing a tooth and not rehabilitate	Triangular	0.07	0.11
Utility periodontitis	Triangular	0.67	0.89
Utility after periodontal treatment	Triangular	0.76	0.96
Utility maintenance therapy	Triangular	0.85	0.98
Utility of tooth loss and no rehabilitation	Triangular	0.55	0.67
Utility tooth loss and removable partial prothesis	Triangular	0.62	0.76

State	Distribution	Mean of logRR^a^	Standard Deviation of logRR^a^
Risk of tooth loss for former smokers	Normal	0.14	0.28
Risk of tooth loss for current smokers	Normal	0.96	0.27

^a^logRR: logarithmic of relative risk

^b^Including training [34]—For the sensitivity analysis, the global cost/program/year was considered per patient to project a conservative impact of including the therapy in the Public Health System.

^c^Used in deterministic sensitivity analysis. Value extracted from Nohlert E, Tegelberg A, Tillgren P, Johansson P, Rosenblad A, Helgason AR. Comparison of a high and a low intensity smoking cessation intervention in a dentistry setting in Sweden: a randomized trial. BMC Public Health. 2009 Apr 30;9:121. 10.1186/1471-2458-9-121

All costs are in U$ dollar

BR (Brazil); WW (Worldwide)

#### Deterministic sensitivity analysis

We varied SCT costs to cover different scenarios, such as different protocols. We based the costs on the Mendes et al. (2016) study [34]. We considered personnel training in a separate analysis as a global cost for SCT program maintenance (Table 3). The projected costs of periodontal treatment, maintenance therapy, and rehabilitation varied by 10%.

The probability of annual tooth loss in non-smokers varied. As we assumed population-based data from non-smokers to be used in the base-case, we tested this assumption using probabilities from studies with Brazilian subjects [37, 38] and also international practice- and university-based data (Additional file 3). The probability of losing a tooth and not receiving rehabilitation varied, with the insertion of a higher probability of any rehabilitation [39].

To observe the influence of the rehabilitation with implant therapy, the cost and utility of this therapy were inserted in the model in the condition of subjects that lose a tooth and received rehabilitation (Table 2).

Utility scores varied using the standard deviation of the mean scores. The discount rate ranged from 3 to 7%.

#### Probabilistic sensitivity analysis

Distribution of variables that could interfere in the model and their respective distributions were inserted in the probabilistic sensitivity analysis (Table 3). Monte Carlo simulations repeated 1000 times were plotted on the cost-effectiveness plane for both analyses (CEA and CUA).

The probability of tooth loss in smokers and non-smokers varied using the natural logarithm of the confidence intervals (CI) of the risk ratios of tooth loss in former and current smokers.

Probability of losing a tooth and not receiving rehabilitation varied between the average CI of this probability.

#### Analysis of uncertainty and cost-acceptability curves

All stochastic model input parameters were expressed using probability distributions derived primarily from the selected studies (Table 1). Modeling assumptions were varied through a series of deterministic sensitivity analyses on the probabilistic model. The assumed probability distributions used for each stochastic model input parameter are presented in Table 3. Normal distributions were assumed for risk of tooth loss, triangular distributions were used for cost and utility variables and a uniform distribution was used in the discount rate.

Average costs, effects, cost-effectiveness, and cost-utility results were based on means of the simulated results. These results were plotted in a cost-effectiveness plane, presenting information on the joint distribution of incremental cost and incremental effectiveness. Therefore, probabilities of combing outcomes (risks and benefits) in different quadrants could be explored.

In Brazilian guidelines for health technology assessments, there is not a threshold to determine whether an intervention is cost-effective or not. Additionally, we considered health effects whose potential willingness to pay (WTP) were not known. This is the reason why Cost-Effectiveness Acceptability Curves (CEAC) were plotted supposing different hypothetical values for WTP thresholds. We did not assume a fixed WTP value and plotted the probability of being cost-effective in different hypothetical WTP values on curves. Then, we aimed to permit health system managers to choose (or not) the intervention depending on their WTP for it.

## Results

### Incremental costs and effects

Implementation of smoking cessation therapy was dominant (less costly and more effective) over non-implementation in both analyses, when the base-case was assumed (Table 4). Implementing the therapy saved approximately US$ 100 over the time horizon accompanied by a slightly better effect, both in CEA and CUA (Table 4).

**Table 4:** Incremental costs and effects for implementation of SCT over the non-implementation of SCT

	Deterministic models			
	Cost	Incremental Costs	Effectiveness^a^	Incremental effects
**Cost-effectiveness analysis**				
Implementation of SCT	3755		55.18	
Non-implementation of SCT	3852		54.60	
		− 97.0		0.58

	Cost	Incremental Costs	Effectiveness^b^	Incremental effects
**Cost-utility analysis**				
Implementation of SCT	3755		49.07	
Non-implementation of SCT	3852		48.57	
		− 97.0		0.50

	Probabilistic models			
	Cost	Incremental Costs	Effectiveness^a^	Incremental effects
**Cost-effectiveness analysis (Mean; 95%CI)**				
Implementation of SCT	3536.53 (2884.95 to 4030.02)		50.22 (39.80 to 57.34)	
Non-implementation of SCT	3022.09 (1954.41 to 3886.84)		42.31 (26.50 to 54.88)	
		514.45 (50.98 to 973.36)		7.91 (2.53 to 13.74)

	Cost		Effectiveness^b^	
**Cost-utility analysis (Mean; 95%CI)**				
Implementation of SCT	3539.36 (2932.78 to 4053.46)		45.05 (36.76 to 51.67)	
Non-implementation of SCT	3494.12 (2763.10 to 4116.78)		43.82 (34.05 to 51.43)	
		45.16 (− 147.96 to 265.55)		1.21 (0.13 to 3.06)

^a^Avoidance, prevention of tooth loss (1 tooth per patient)

^b^Oral-related quality-adjusted years (QALY)

CI, confidence interval

### Sensitivity analyses and characterization of uncertainty

**Fig. 2:**
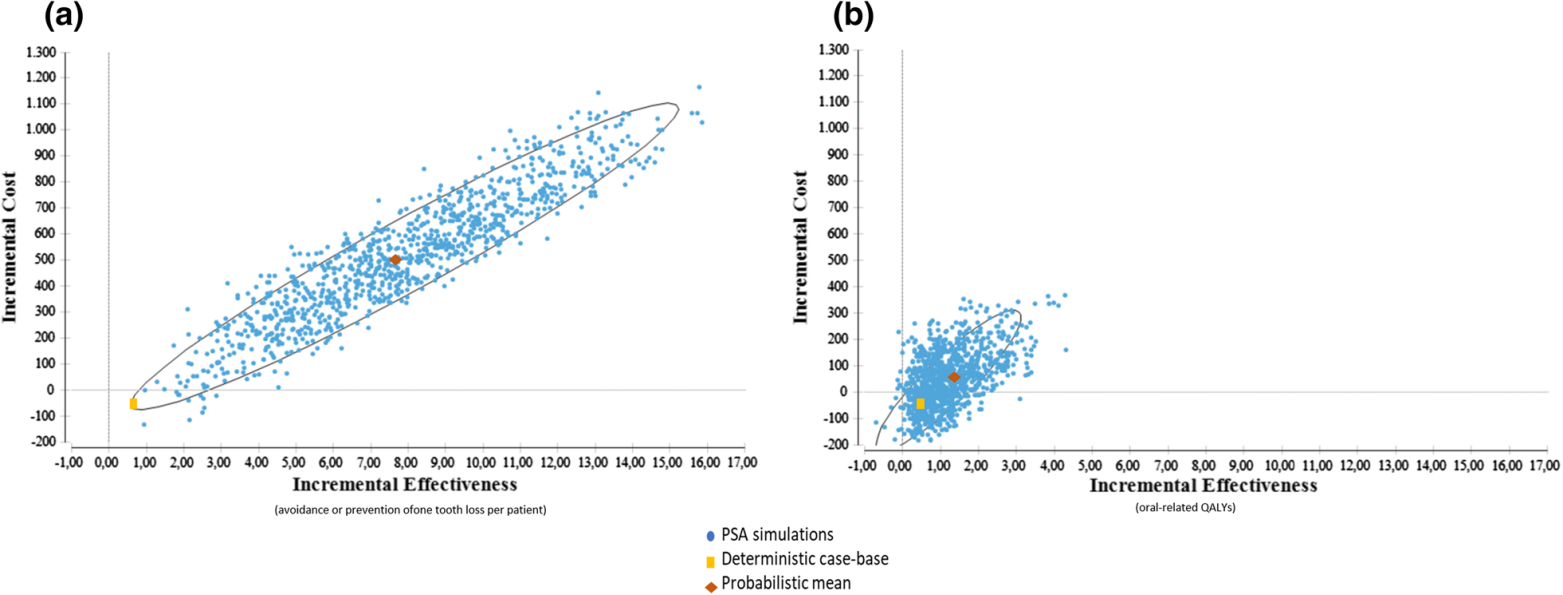
Cost-effectiveness planes considering as health effects **a** the avoidance or prevention of one tooth loss per patient and **b** the oral-related quality-adjusted life-years. (P_quadrant:_ probability of simulated points is found on that quadrant—NE: Northest, NW: Northwest, SE: Southest; SW: southwest, PSA: Probabilistic Simulation Analysis)

The majority of the parameters tested in sensitivity analyses did not impact the results. Thus, implementation of SCT remained cost-effective despite varying some model assumptions. When we varied the costs of SCT to cover different scenarios, implementation of the therapy was also not dominant over non-implementation for some of the scenarios (Fig. 2). We observed, on average, a Δcost = U$201 for implementing SCT when the maximum scenario was considered). In this case, incremental costs of US$ 347 per tooth loss avoided and US$ 403 per oral-QALY gained were calculated. A similar trend was observed when we tested a much lower probability of smoking cessation observed in a non-population based study [40], resulting in a still better effect of SCT, increasing on average 100 dollars per patient at the adopted time horizon.

Importantly, even when we considered implant therapy in the rehabilitation of patients with tooth loss, implementation of SCT remained cost-effective and non-implementation of SCT was dominated by the implementation. Other assumptions did not influence previous trends.

When considering the uncertainties, implementation of SCT tended to be more costly and more effective in the great majority of the simulated cases to the CEA (99.9%). In the CUA, 35% of the simulated cases were in the dominant quadrant (Fig. 2). Besides, approximately 64% of the cases would be in the northeast quadrant, being maximum incremental cost as high as approximately US$350 associated with incremental oral-related QALY gained ranging from 0 to 4.5 (Fig. 2 and Additional file 2).

**Fig. 3:**
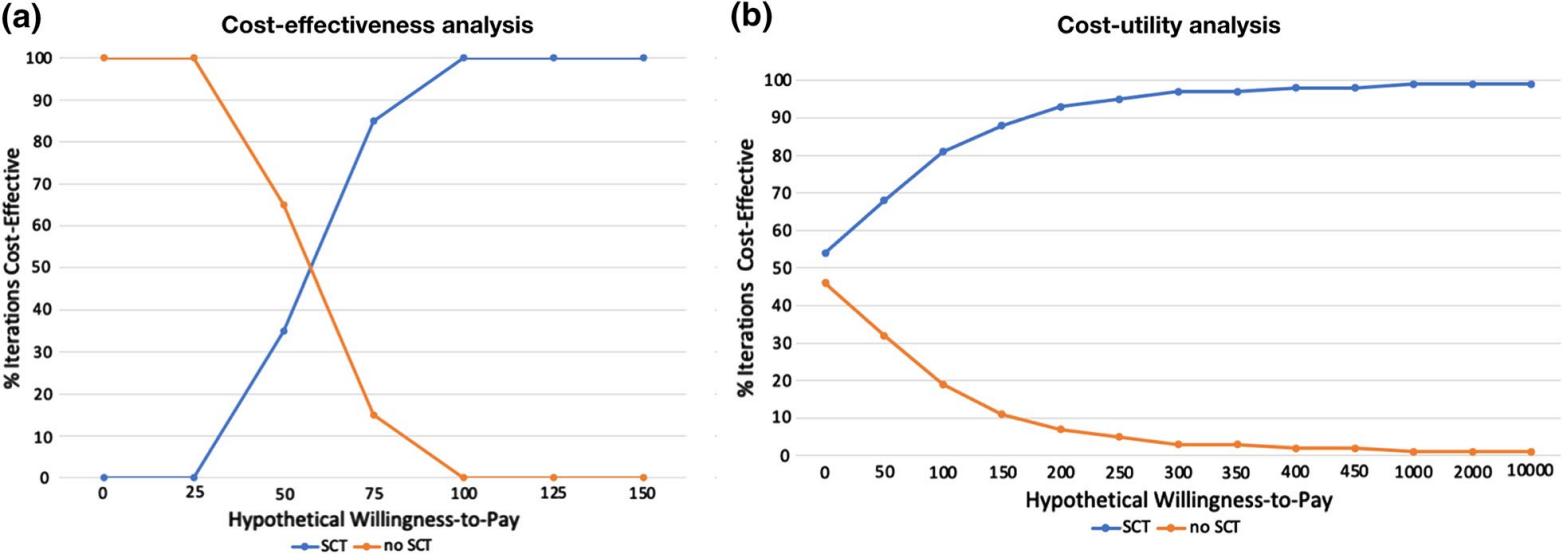
Acceptability curves considering as health effects **a** the avoidance or prevention of tooth loss and **b** the oral-related quality-adjusted life-years. We considered different hypothetical willingness-to-pay (WTP) values and, for each them, we plotted the probability of being a cost-effective option in order to permit health system managers to choose (or not) the intervention depending on their own WTP for it

CEACs demonstrated the variations in probability of SCT being a cost-effective option vs. non-implementation for different WTP thresholds. At a hypothetical WTP of US$ 100, SCT implementation, is considered as the optimal strategy in 99% of cases, when concerning tooth loss (Fig. 3). When oral-related QALYs were considered for the analyses, at the same hypothetical WTP, 79% of the iterations would be cost-effective (Fig. 3). Still considering the QALYs, 98% of cost-effective iterations were observed at a WTP = US$450.

## Discussion

The results of this study show that implementation of SCT is an efficient way of allocating resources compared to its non-implementation, in the perspective of the Brazilian PHS. Even considering additional costs related to cognitive-behavioral intervention for smoking cessation, they tended to be favorably balanced by the health gain achieved. In some circumstances, the initial extra expenses in implementing the SCT may be compensated by costs saved in the subsequent dental treatment for smokers.

We chose the PHS perspective because all Brazilian citizens are entitled to the services provided by the public system [41]. Currently, SCT in the PHS is conducted by a multi-professional team that does not include dentists. However, this intervention could be delivered by dentists in the Brazilian PHS [42], especially if one considers that SCT conducted by oral health professionals increases tobacco abstinence rates [43].

The cost-effectiveness of smoking on periodontal therapy in private practice was evaluated by Fardal et al. [7], based on costs of the American Dental Association. They found an increased cost of periodontal treatment for smokers and that the lifetime cost of periodontal therapy is equivalent to about 25% of the cost of smoking for patients who smoke 20 cigarettes per day. However, the authors did not evaluate the cost-effectiveness of SCT. Feldman et al. [44] compared the cost-effectiveness of a high-intensity therapy with a low-intensity smoking cessation intervention in a Swedish dental setting. Their results favored the high-intensity smoking cessation intervention when willingness to pay was €4000/QALY. The utility weights were derived via general health-related quality of life questionnaire. Their results are important because this high-intensity smoking cessation intervention is very similar to SCT adopted in Brazilian PHS. According to their findings and our results, SCT is a cost-effective intervention for periodontitis patients, which empathizes the necessity to increase application of SCT in the Brazilian PHS dental setting.

From the Brazilian PHS perspective, SCT could be considered a cost-effective option (probability from 79–99%) even considering a low WTP threshold (US$), as we assumed to exemplify. Even when uncertainties are considered, we could observe that a low incremental cost (not exceeding US$350) could be expected. Since we do not have a known WTP for the health effects considered as outcomes in the present evaluations, we analyzed the CEA and the probabilities of simulated cases yield on different quadrants to permit the decision-maker to judge this information and consider if it is acceptable. It is possible to opt for the different preferences for inefficiencies occurring in different quadrants since both size and nature of risks may be presented [45].

Although in both analyses, CEA and CUA, the implementation of SCT was cost-effective, the health effects in CUA were smaller than in CEA. This result was expected because effects in subjective measures are less evident than in objective measures.

We chose tooth loss as the outcome of CEA because it is considered the true endpoint of periodontal disease [46] and the most important objective outcome to the patient [47]. Therefore, tooth loss should be considered the most appropriate outcome in an economic analysis [31]. Some studies used surrogate outcomes, such as bleeding on probing, plaque index, probing depth reduction and clinical attachment gain [48–50]. However, the precise impact of these surrogate outcomes on the patient is unclear [31].

We included a CUA in our study because this type of analysis allows including a measure of the patients’ preferences and perception about their health. The importance of patient-related outcomes is impacting more studies with CUA in dentistry [51]. However, there is still a scarcity of this type of analysis in some regions, such as in South American countries [51]. Medical literature usually uses EuroQOL-5Dimension (EQ-5D) [52] or Structured Form 6 Dimension (SF-6D) [53] to determine QALY. However, these instruments evaluate general health, which is unlikely to be sensitive to important domains of oral health, such as chewing ability or aesthetics [54]. In the absence of a suitable measure in Dentistry, and considering the low sensitivity of medical questionnaires, we assumed that the utility was proportional to quality of life. This methodology was proposed by previous studies that converted scores from quality of life questionnaires to utility scores between 0 and 1 and reflected changes related to periodontal conditions/treatment [55, 56]. Even though this is not the ideal approach, this interim tool allowed the incorporation of a patient-centered approach into the analysis. We believe that these limitations do not impact ur findings because the utility scores were used in both analysed strategies.

The inclusion of CUA also allows verifying that there are situations in which SCT could be cost-saving compared to its non-implementation, which is an additional argument to endorse this therapy in the PHS. On the other hand, our CUA should be interpreted carefully, because it is not possible to affirm that quality of life has a linear relationship with the utility. We expect that oral health-related quality of life instruments may have registered oral health conditions that are important to the patient, which are not necessarily comparable to general health status. Oral health-specific utility measures are probably more sensitive in capturing the effectiveness of oral health interventions [50]. Instruments that use an indexed scale for oral health-related aspects need to be developed to improve the comparison between studies and different therapies.

We applied sensitivity analysis to characterize the uncertainty of our results. We tested a model with implants because this type of rehabilitation presents better utility scores than a partial prosthesis, but it also includes higher costs in the model. However, even considering these higher costs, rehabilitation with implants did not affect the results and the implementation of SCT remained a cost-effective therapy. Further, it was dominant over the non-implementation of SCT. We did not test partial fixed prostheses in our models since they are not available in the Brazilian PHS. Moreover, they may result in additional harm such as pulp exposure, which may lead to endodontic treatment [18].

We considered different scenarios of the use of resources in SCT [34] when the SCT costs were varied in the sensitivity analysis. We observed a marginal dominance, since when varying the SCT costs, the therapy remained cost-effective (but not dominant, as in the base case). These findings also provide evidence that SCT should be implemented for PHS. Since cognitive-behavioural therapy is responsible for most SCT costs, we believe the variation in SCT costs considering these different scenarios may also reflect possible variations proposed in different protocols for SCT, even those different from Brazil. Although the costs for professional training were not included in the primary model, even under a conservative approach, these additional costs did not impact the results.

The number of sessions of cognitive-behavioral therapy in SCT seems to exert the same influence on the cost-effectiveness of the therapy. The number and frequency of periodontal maintenance sessions can vary according to clinical conditions, such as extension and severity of the disease. The number of SCT sessions was fixed, as in the Brazilian protocol for SCT, but different scenarios were used in the sensitivity analyses to explore these possible variations. Even when we tested variations in SCT costs, the implementation of SCT remained cost-effective. Therefore, despite exploring possible variables and uncertainties related to our models for CEA and CUA, we reinforce that the SCT may be a cost-effective therapy for periodontitis patients to be implemented in the Brazilian PHS.

Economic evaluations are a standard tool in the assessment of health care technologies to maximize benefits from the available resources [57]. The need to allocate public finances increased the interest in cost-effectiveness research in dentistry [58]. A cost-effectiveness criterion can play an important role by guiding the incorporation of new technologies into the population. Policymakers from some countries, such as Australia, Canada and European countries, have adopted economic evaluations to their drug guidelines and reimbursement [59]. The present findings are especially important for the Brazilian PHS and demonstrate that SCT should be implemented. It is necessary to emphasize that studies with different populations, costs and perspectives should be conducted to confirm the cost-effectiveness of the implementation of SCT concerning tooth loss in different scenarios. This model can be used as a model for future cost-effectiveness analysis with costs and effects from other countries. As we adopted a model in which repetitions of SCT were not included due to smoking relapse, other models may also test the influence of variables related to that in the cost-effectiveness of SCT.

## Conclusions

Implementation of SCT in periodontitis patients from the Brazilian public health system (PHS) is an efficient way of allocating resources compared to its non-implementation, possibly to be more effective and also cost-saving in some circumstances both when considering tooth loss or oral health-related QALY gains.

## Supplementary Information

**Additional file 1**. Utility scores.

**Additional file 2**. Expected costs, outcomes, cost-effectiveness, cost-utility based on deterministic model by varying each variable from a minimum (green) to a maximum (red) values.

**Additional file 3**. Meta-analysis of 1-year probabilities of tooth loss in non-smokers.

**Additional file 4**. References used in sensitivity analysis.

## Data Availability

All data generated or analyzed during this study are included within the article (and its additional files).

## Abbreviations

SCT: Smoking cessation therapy
CHEERS: Consolidated Health Economic Evaluation Reporting Standards
PHS: Brazilian public health system
CEA: Cost-effectiveness analysis
CUA: Cost-utility analysis
QALY: Quality-adjusted life years
OHIP: Oral Health Impact Profile
CEOs: Dental Specialty Centers
PPP: Purchasing power parity
WTP: Potential willingness to pay
CEAC: Cost-Effectiveness Acceptability Curves

## References

[1] World Health Organization. WHO report on the global tobacco epidemic 2017. 2017. Available from: https://www.who.int/tobacco/global_report/2017/en/

[2] Naseri R, Yaghini J, Feizi A (2020). Levels of smoking and dental implants failure: A systematic review and meta-analysis. J Clin Periodontol.

[3] Helal O, Göstemeyer G, Krois J, Fawzy El Sayed K, Graetz C, Schwendicke F (2019). Predictors for tooth loss in periodontitis patients: Systematic review and meta-analysis. J Clin Periodontol.

[4] Zhang Y, He J, He B, Huang R, Li M (2019). Effect of tobacco on periodontal disease and oral cancer. Tob Induc Dis.

[5] Ide R, Hoshuyama T, Wilson D, Takahashi K, Higashi T (2009). The effects of smoking on dental care utilization and its costs in Japan. J Dent Res.

[6] Park YD, Kang JO, Kim SJ, Kwon HJ, Hwang JH, Hwang KS (2012). Estimation of the costs of smoking-related oral disease: a representative South Korean study. Int Dent J.

[7] Fardal Ø, Grytten J, Martin J, Ellingsen S, Fardal P, Heasman P, Linden GJ (2018). Adding smoking to the Fardal model of cost-effectiveness for the lifetime treatment of periodontal diseases. J Periodontol.

[8] Bai JW, Chen XX, Liu S, Yu L, Xu JF (2017). Smoking cessation affects the natural history of COPD. Int J Chron Obstruct Pulmon Dis.

[9] Jha P, Ramasundarahettige C, Landsman V, Rostron B, Thun M, Anderson RN, McAfee T, Peto R (2013). 21st-century hazards of smoking and benefits of cessation in the United States. N Engl J Med.

[10] Preshaw PM, Heasman L, Stacey F, Steen N, McCracken GI, Heasman PA (2005). The effect of quitting smoking on chronic periodontitis. J Clin Periodontol.

[11] Rosa EF, Corraini P, de Carvalho VF, Inoue G, Gomes EF, Lotufo JP, De Micheli G, Pannuti CM (2011). A prospective 12-month study of the effect of smoking cessation on periodontal clinical parameters. J Clin Periodontol.

[12] Dietrich T, Walter C, Oluwagbemigun K, Bergmann M, Pischon T, Pischon N, Boeing H (2015). Smoking, smoking cessation, and risk of tooth loss: the EPIC-potsdam study. J Dent Res.

[13] Souto MLS, Rovai ES, Villar CC, Braga MM, Pannuti CM (2019). Effect of smoking cessation on tooth loss: a systematic review with meta-analysis. BMC Oral Health.

[14] Sanz M, Herrera D, Kebschull M, Chapple I, Jepsen S, Beglundh T, Sculean A, Tonetti MS (2020). EFP Workshop Participants and Methodological Consultants. Treatment of stage I-III periodontitis-The EFP S3 level clinical practice guideline. J Clin Periodontol.

[15] Kahende JW, Loomis BR, Adhikari B, Marshall L (2009). A review of economic evaluations of tobacco control programs. Int J Environ Res Public Health.

[16] Ruger JP, Lazar CM (2012). Economic evaluation of pharmaco- and behavioral therapies for smoking cessation: a critical and systematic review of empirical research. Annu Rev Public Health.

[17] Gaunt F, Devine M, Pennington M, Vernazza C, Gwynnett E, Steen N, Heasman P (2008). The cost-effectiveness of supportive periodontal care for patients with chronic periodontitis. J Clin Periodontol.

[18] Schwendicke F, Stolpe M, Plaumann A, Graetz C (2016). Cost-effectiveness of regular versus irregular supportive periodontal therapy or tooth removal. J Clin Periodontol.

[19] Solowiej-Wedderburn J, Ide M, Pennington M (2017). Cost-effectiveness of non-surgical periodontal therapy for patients with type 2 diabetes in the UK. J Clin Periodontol.

[20] Tay JRH, Ng E, Nair R, Tan ZS, Tan SHX (2021). Economic evaluations in the treatment and evaluation of patients with periodontal disease: a critical review. J Clin Periodontol..

[21] Gafini A (1996). Economic evaluation of health care interventions: an economist's perspective. ACP J Club.

[22] Husereau D, Drummond M, Petrou S, Carswell C, Moher D, Greenberg D, Augustovski F, Briggs AH, Mauskopf J, Loder E; CHEERS Task Force. Consolidated Health Economic Evaluation Reporting Standards (CHEERS) statement. BMJ. 2013;346:f1049. 10.1136/bmj.f1049 23529982

[23] IBGE. Tábuas Completas de Mortalidade. 2017. Available from: https://www.ibge.gov.br/estatisticas/sociais/populacao/9126-tabuas-completas-de-mortalidade.html?&t=downloads.

[24] Caton JG, Armitage G, Berglundh T, Chapple ILC, Jepsen S, Kornman KS, Mealey BL, Papapanou PN, Sanz M, Tonetti MS (2018). A new classification scheme for periodontal and peri-implant diseases and conditions—introduction and key changes from the 1999 classification. J Clin Periodontol.

[25] Tonetti MS, Greenwell H, Kornman KS. Staging and grading of periodontitis: framework and proposal of a new classification and case definition. J Clin Periodontol. 2018;45 Suppl 20:S149-S161. 10.1111/jcpe.12945. Erratum in: J Clin Periodontol. 2019 Jul;46(7):787. 10.1111/jcpe.12945 29926495

[26] Rosa EF, Corraini P, Inoue G, Gomes EF, Guglielmetti MR, Sanda SR, Lotufo JP, Romito GA, Pannuti CM (2014). Effect of smoking cessation on non-surgical periodontal therapy: results after 24 months. J Clin Periodontol.

[27] Brasil. Ministério da Saúde. Secretaria de Ciência T e IED de C e T. Diretrizes metodológicas: diretriz de avaliação econômica. 2014. Availabe from: bvsms.saude.gov.br/bvs/publicacoes/diretrizes_metodologicas_diretriz_avaliac ao_economica.pdf

[28] Prado GF, Lombardi EM, Bussacos MA, Arrabal-Fernandes FL, Terra-Filho M, Santos UP (2011). A real-life study of the effectiveness of different pharmacological approaches to the treatment of smoking cessation: re-discussing the predictors of success. Clinics (Sao Paulo).

[29] Zhu S, Melcer T, Sun J, Rosbrook B, Pierce JP (2000). Smoking cessation with and without assistance: a population-based analysis. Am J Prev Med.

[30] Pereira, A. C., Vieira, V., & Frias, A. C. 2016. *SB São Paulo Pesquisa Estadual de Saúde Bucal*. Águas de São Pedro.

[31] Vernazza C, Heasman P, Gaunt F, Pennington M. How to measure the cost-effectiveness of periodontal treatments. Periodontol 2000. 2012;60(1):138–46. 10.1111/j.1600-0757.2011.00406.x 22909111

[32] Slade GD, Spencer AJ (1994). Development and evaluation of the Oral Health Impact Profile. Community Dent Health.

[33] Pires CP, Ferraz MB, de Abreu MH (2006). Translation into Brazilian Portuguese, cultural adaptation and validation of the oral health impact profile (OHIP-49). Braz Oral Res.

[34] Mendes AC, Toscano CM, Barcellos RM, Ribeiro AL, Ritzel JB, Cunha VS, Duncan BB (2016). Costs of the smoking cessation program in Brazil. Rev Saude Publica.

[35] de Oliveira RS, de Morais HMM, de Goes PSA, Botazzo C (2015). Magalhães BG Relações contratuais e perfil dos cirurgiões dentistas em centros de especialidades odontológicas de baixo e alto desempenho no Brazil. Saude e Sociedade.

[36] Brasil. Ministério da Saúde. Banco de Preços em Saúde. 2017. Availabe from: http://portalms.saude.gov.br/gestao-do-sus/economia-da-saude/banco-de-precos-em-saude

[37] Chambrone LA, Chambrone L (2006). Tooth loss in well-maintained patients with chronic periodontitis during long-term supportive therapy in Brazil. J Clin Periodontol.

[38] Stadler AF, Mendez M, Oppermann RV, Gomes SC (2017). Tooth loss in patients under periodontal maintenance in a private practice: a retrospective study. Braz Dent J.

[39] Brasil. Ministério da Saúde. SB Brasil 2010: Pesquisa Nacional de Saúde Bucal: resultados principais. 2012. Available from: 10.3310/hta21210

[40] Pretzl B, Sayed S, Weber D, Eickholz P, Baumer A (2018). Tooth loss in periodontally compromised patients: results 20 years after active periodontal therapy. J Clin Periodontol.

[41] Castro MC, Massuda A, Almeida G, Menezes-Filho NA, Andrade MV, de Souza Noronha KVM, Rocha R, Macinko J, Hone T, Tasca R, Giovanella L, Malik AM, Werneck H, Fachini LA, Atun R (2019). Brazil's unified health system: the first 30 years and prospects for the future. Lancet.

[42] Pucca GA, Gabriel M, de Araujo ME, de Almeida FC (2015). Ten years of a national oral health policy in Brazil: innovation, boldness, and numerous challenges. J Dent Res.

[43] Holliday R, Hong B, McColl E, Livingstone-Banks J, Pershaw P, Carr AB, Ebbert J (2021). Interventions for tobacco cessation delivered by dental professionals. Cochrane Database Syst Rev.

[44] Feldman I, Helgason AR, Johansson P, Tegelberg Å, Nohlert E (2019). Cost-effectiveness of a high-intensity versus a low-intensity smoking cessation intervention in a dental setting: long-term follow-up. BMJ Open.

[45] Sendi P, Gafni A, Birch S (2002). Opportunity costs and uncertainty in the economic evaluation of health care interventions. Health Econ.

[46] Hujoel PP, DeRouen TA (1995). A survey of endpoint characteristics in periodontal clinical trials published 1988–1992, and implications for future studies. J Clin Periodontol.

[47] Pannuti CM, Sendyk DI, GraÇas YTD, Takai SL, SabÓia VPA, Romito GA, Mendes FM (2020). Clinically relevant outcomes in dental clinical trials: challenges and proposals. Braz Oral Res.

[48] Listl S, Tu YK, Faggion CM (2010). A cost-effectiveness evaluation of enamel matrix derivatives alone or in conjunction with regenerative devices in the treatment of periodontal intra-osseous defects. J Clin Periodontol.

[49] Jönsson B, Ohrn K, Lindberg P, Oscarson N (2012). Cost-effectiveness of an individually tailored oral health educational programme based on cognitive behavioural strategies in non-surgical periodontal treatment. J Clin Periodontol.

[50] Mohd-Dom TN, Wan-Puteh SE, Muhd-Nur A, Ayob R, Abdul-Manaf MR, Abdul-Muttalib K, Aljunid SM (2014). Cost-effectiveness of periodontitis management in public sector specialist periodontal clinics: a societal perspective research in Malaysia. Value Health Reg Issues.

[51] Hettiarachchi RM, Kularatna S, Downes MJ, Byrnes J, Kroon J, Lalloo R, Johnson NW, Scuffham PA (2018). The cost-effectiveness of oral health interventions: a systematic review of cost-utility analyses. Community Dent Oral Epidemiol.

[52] Brooks R (1996). EuroQol: the current state of play. Health Policy.

[53] Brazier JE, Harper R, Jones NM, O'Cathain A, Thomas KJ, Usherwood T, Westlake L (1992). Validating the SF-36 health survey questionnaire: new outcome measure for primary care. BMJ.

[54] Stone SJ, McCracken GI, Heasman PA, Staines KS, Pennington M (2013). Cost-effectiveness of personalized plaque control for managing the gingival manifestations of oral lichen planus: a randomized controlled study. J Clin Periodontol.

[55] Korenori A, Koji K, Yuki T, Murata T, Sachiko TM, Shunsuke B (2018). Cost-effectiveness of molar single-implant versus fixed dental prosthesis. BMC Oral Health.

[56] Mohd-Dom T (2014). Quality-adjusted tooth years (QATY) as an outcome measure of periodontal treatment. BMC Public Health.

[57] Listl S, Grytten JI, Birch S (2019). What is health economics?. Community Dent Health.

[58] Eow J, Duane B, Solaiman A, Hussain U, Lemasney N, Ang R, O'Kelly-Lynch N, Girgis G, Collazo L, Johnston B (2019). What evidence do economic evaluations in dental care provide?. A scoping review Community Dent Health.

[59] Neumann PJ, Johannesson M. From principle to public policy: using cost-effectiveness analysis. Health Aff (Millwood). 1994 Summer;13(3):206–14. 10.1377/hlthaff.13.3.206 7927151

[60] Fardal Ø, Johannessen AC, Linden GJ (2004). Tooth loss during maintenance following periodontal treatment in a periodontal practice in Norway. J Clin Periodontol.

