# Supplementary material for: Smoking Cessation therapy is a cost-effective intervention to avoid tooth loss in Brazilian subjects with periodontitis: an economic evaluation
Source: BMC Oral Health. 2021 Dec 3;21:616. doi: 10.1186/s12903-021-01932-2 (PMC8642876; doi:10.1186/s12903-021-01932-2)
Supplement: Supplementary file 1 — Additional file 1. Utility scores. [file 12903_2021_1932_MOESM1_ESM.docx]

Supplementary file 01: Utility scores

| **Outcome** | **Data source** | **OHIP score** | **Utility score** |
| --- | --- | --- | --- |
| Periodontitis | Mendez et al. 2017 | OHIP-14: 17.3 | 0.76 |
| After periodontal treatment | Mendez et al. 2017 | OHIP-14: 9.5 | 0.86 |
| Maintenance therapy | Mendez et al. 2018 | OHIP-14: 4.54 | 0.93 |
| Tooth loss and no rehabilitation | Barreto et al. 2011 | OHIP-49: 125.9 | 0.61 |
| Tooth loss and removable partial prothesis | Barreto et al. 2011 | OHIP-49: 88.6 | 0.69 |
| Tooth loss and implant therapy | Goiato et al. 2015 | 0HIP-14: 18.8 | 0.75 |
