# Supplementary material for: Smoking Cessation therapy is a cost-effective intervention to avoid tooth loss in Brazilian subjects with periodontitis: an economic evaluation
Source: BMC Oral Health. 2021 Dec 3;21:616. doi: 10.1186/s12903-021-01932-2 (PMC8642876; doi:10.1186/s12903-021-01932-2)
Supplement: Supplementary file 2 — Additional file 2. Expected costs, outcomes, cost-effectiveness, cost-utility based on deterministic model by varying each variable from a minimum (green) to a maximum (red) values. [file 12903_2021_1932_MOESM2_ESM.docx]

Supplementary file 2: Expected costs, outcomes, cost-effectiveness, cost-utility based on deterministic model by varying each variable from a minimum (green) to a maximum (red) value

| **Variables** | **Value** | **Incremental Cost ($)** | **Incremental Effectiveness§** | **Incremental**  **QALYs** |
| --- | --- | --- | --- | --- |
| Cost of maintenance therapy in smokers | 94.78 | -28 | 0.58 | 0.50 |
|  | 115.86 | -166 |  |  |
| Cost of maintenance therapy in former smokers | 47.39 | -133 | 0.58 | 0.50 |
|  | 57.93 | -61 |  |  |
| Cost of SCT | 185.86 | -138 | 0.58 | 0.50 |
|  | 355.73 | -32 |  |  |
|  | 525.59 | 201 |  |  |
| Probability of cessation with any therapy* | 0.16# | 101 | 0.23 | 0.20 |
|  | 0.23 | -12 | 0.43 | 0.35 |
|  | 0.38 | -209 | 0.78 | 0.67 |
| Probability of tooth loss in non-smokers | 0.0003 | -148 | 0.06 | 0.05 |
|  | 0.005 | -63 | 0.91 | 0.78 |
|  | 0.006 | -47 | 1.06 | 0.41 |
|  | 0.09 | 253 | 1.93 | 1.72 |
| Relative risk for tooth loss in former smokers | 0.95 | -92 | 0.70 | 0.60 |
|  | 1.35 | -101 | 0.46 | 0.40 |
| Relative risk for tooth loss in current smokers | 2.29 | -92 | 0.65 | 0.56 |
|  | 2.96 | -98 | 1.02 | 0.88 |
|  | 4.17 | -28 | 1.64 | 1.41 |
| Probability of losing a tooth and not rehabilitate | 0.00 | 102 | 0.58* | 0.46 |
|  | 0.41 | 79 | 0.58* | 0.64 |
| Discount rate | 1.03 | -91 | 0.58 | 0.50 |
|  | 1.07 | -103 |  |  |
| Utility periodontitis | 0.67 | -97 | ----- | 0.50* |
|  | 0.89 |  |  | 0.50* |
| Utility after periodontal treatment | 0.76 | -97 | ----- | 0.47 |
|  | 0.96 |  |  | 0.53 |
| Utility maintenance therapy | 0.85 | -97 | ----- | 0.48 |
|  | 0.98 |  |  | 0.51 |
| Utility tooth loss and no rehabilitation | 0.55 | -97 | ----- | 0.50* |
|  | 0.67 |  |  | 0.50* |
| Utility tooth loss and removable partial prosthesis | 0.62 | -97 | ----- | 0.50* |
|  | 0.76 |  |  | 0.50* |
| *Although category values have been varied, there was no clinically relevant change in effectiveness values probably due to the small differences between both extremes (differences could be probably observed at level of many decimals- that is why they were not considered clinically relevant in this case)  ǂvalue tested in deterministic sensitivity analysis just to explore a different context – study including much older patients in comparison to base-case.  § avoidance or prevention of tooth loss  QALY: Quality-adjusted life years | | | | |
