# Supplementary figures and images for: Smoking Cessation therapy is a cost-effective intervention to avoid tooth loss in Brazilian subjects with periodontitis: an economic evaluation
Source: BMC Oral Health. 2021 Dec 3;21:616. doi: 10.1186/s12903-021-01932-2 (PMC8642876; doi:10.1186/s12903-021-01932-2)

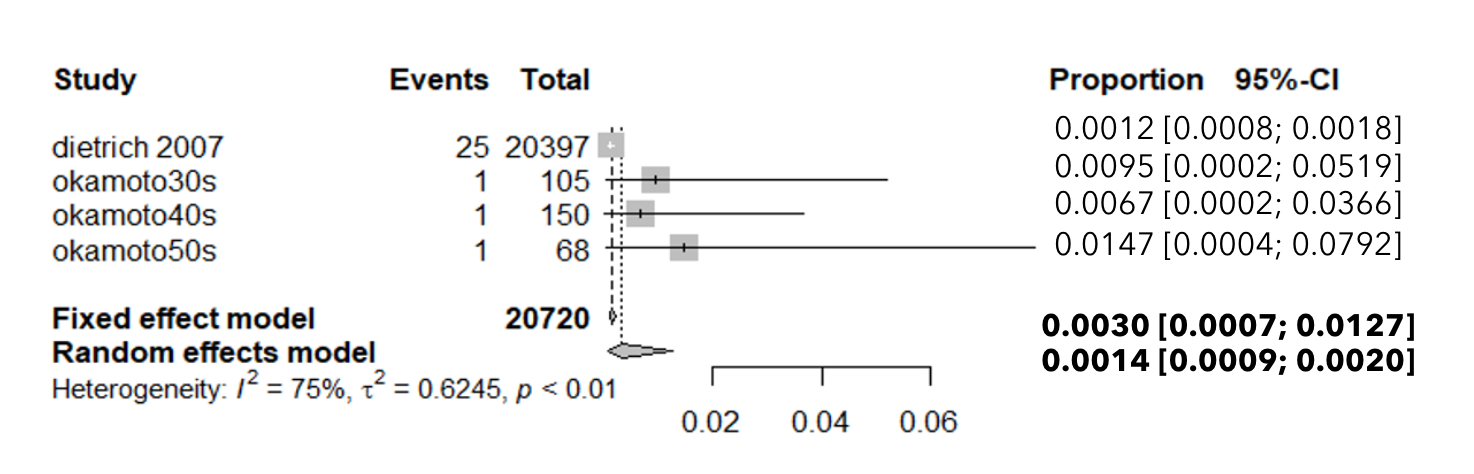

Supplement: Supplementary file 3 — Additional file 3. Meta-analysis of 1-year probabilities of tooth loss in non-smokers. [file 12903_2021_1932_MOESM3_ESM.png]
