# Supplementary material for: Smoking Cessation therapy is a cost-effective intervention to avoid tooth loss in Brazilian subjects with periodontitis: an economic evaluation
Source: BMC Oral Health. 2021 Dec 3;21:616. doi: 10.1186/s12903-021-01932-2 (PMC8642876; doi:10.1186/s12903-021-01932-2)
Supplement: Supplementary file 4 — Additional file 4. References used in sensitivity analysis. [file 12903_2021_1932_MOESM4_ESM.docx]

Chambrone LA, Chambrone L. Tooth loss in well-maintained patients with chronic periodontitis during long-term supportive therapy in Brazil. J Clin Periodontol. 2006 Oct;33(10):759-64. doi: 10.1111/j.1600-051X.2006.00972.x.

Fardal Ø, Johannessen AC, Linden GJ. Tooth loss during maintenance following periodontal treatment in a periodontal practice in Norway. J Clin Periodontol. 2004; 31: 550-555. doi: 10.1111/j.1600-051X.2004.00519.x.

Stadler AF, Mendez M, Oppermann RV, Gomes SC. Tooth Loss in Patients under Periodontal Maintenance in a Private Practice: A Retrospective Study. Braz Dent J. 2017 Jul-Aug;28(4):440-446. doi: 10.1590/0103-6440201701476.

Fiore MC, Jaén CR, Baker TB, et al. Treating tobacco use and dependence: 2008 update. Washington (DC): US Department of Health and Human Services, Public Health Service; 2008

Nohlert E, Tegelberg A, Tillgren P, Johansson P, Rosenblad A, Helgason AR. Comparison of a high and a low intensity smoking cessation intervention in a dentistry setting in Sweden: a randomized trial. BMC Public Health. 2009 Apr 30;9:121. doi: 10.1186/1471-2458-9-121

Hirschfeld L, Wasserman B. A long-term survey of tooth loss in 600 treated periodontal patients. J Clinical Periodontol. 1978. doi: [10.1902/jop.1978.49.5.225](https://doi.org/10.1902/jop.1978.49.5.225)

Pretzl B, Sayed S, Weber D, Eickholz P, Baumer A. Tooth loss in periodontally compromised patients: results 20 years after active periodontal therapy. J Clin Periodontol 2018 Nov;45(11):1356-1364. doi: 10.1111/jcpe.13010. Epub 2018 Oct 23.

Pretzl B , Kaltschmitt J, KimT,  Reitmeir P, [Eickholz](https://pubmed.ncbi.nlm.nih.gov/?term=Eickholz+P&cauthor_id=18199151) P. Tooth loss after active periodontal therapy. 2: tooth-related factors. J Clin Periodontol 2008 Feb;35(2):175-82. doi: 10.1111/j.1600-051X.2007.01182.x.

Petsos H, Schacher B, Ramich T, Nickles K, Dannewitz B, Arendt S, Seidel K, Eickholz P. Retrospectively analysed tooth loss in periodontally compromised patients: Long-term results 10 years after active periodontal therapy-Patient-related outcomes. J Periodontal Res. 2020 Dec;55(6):946-958. doi: 10.1111/jre.12786.

Ramseier CA, Suvan JE. Behaviour change counselling for tobacco use cessation and promotion of healthy lifestyles: a systematic review. J Clin Periodontol. 2015 Apr;42 Suppl 16:S47-58. doi: 10.1111/jcpe.12351

Souto MLS, Rovai ES, Villar CC, Braga MM, Pannuti CM. Effect of smoking cessation on tooth loss: a systematic review with meta-analysis. BMC Oral Health. 2019 Nov 12;19(1):245

Dietrich T, Maserejian NN, Joshipura KJ, Krall EA, Garcia RI. Tobacco use and incidence of tooth loss among US male health professionals. J Dent Res. 2007 Apr;86(4):373-7. doi: 10.1177/154405910708600414.

Okamoto Y, Tsuboi S, Suzuki S, Nakagaki H, Ogura Y, Maeda K, Tokudome S. Effects of smoking and drinking habits on the incidence of periodontal disease and tooth loss among Japanese males: a 4-yr longitudinal study. J Periodontal Res. 2006 Dec;41(6):560-6. doi: 10.1111/j.1600-0765.2006.00907.x.
